# Aseptic meningitis in a patient taking etanercept for rheumatoid arthritis: a case report

**DOI:** 10.1186/1757-1626-1-364

**Published:** 2008-12-01

**Authors:** Matthew J Booker, Julia Flint, Shanmugam Saravana

**Affiliations:** 1 Department of Rheumatology, City Hospital, Birmingham, UK

## Abstract

**Background:**

We report a case of a 53 year old lady recently commenced on etanercept, an anti-TNF (tumour necrosis factor) therapy for rheumatoid arthritis presenting with confusion, pyrexia and an erythematous rash.

**Case presentation:**

A lumbar puncture was highly suggestive of bacterial meningitis, but CSF cultures produced no growth, and polymerase chain reactions (PCR) for all previously reported bacterial, fungal and viral causes of meningitis were negative.

**Conclusion:**

This case report describes aseptic meningitis as a previously unreported complication of etanercept therapy, and serves as a reminder of the rare but potentially life-threatening risk of serious infections in patients taking anti-TNF therapy for a variety of conditions.

## Background

Etanercept functions as a soluble tumour necrosis factor receptor, and is effective for the treatment of moderate to severe rheumatoid arthritis [1]. It has also been licensed for use in the treatment of juvenile idiopathic arthritis, psoriatic arthritis, moderate to severe plaque psoriasis and ankylosing spondylitis.

Due to its modulatory effect on the immune system, infections are a predictable potential complication of anti-TNF therapy and patients are closely monitored. Rheumatoid arthritis confers a higher baseline risk infections, and when patients are treated with anti-TNF therapy, the increased risk of infection has been extensively studied and debated. Large cohort studies have shown an increase in the rate of infection in patients on anti-TNF therapy when compared to DMARDs alone [2,3], particularly within the first six months of treatment, but this has not been replicated in all randomised controlled studies and observational cohort studies [4]. Re-activation of TB is a potential side effect of anti-TNF therapy, although has been reported more frequently with infliximab than etanercept. Other reported serious side effects of etanercept include reactivation of hepatitis B, demyelination (new onset or exacerbation), pancytopenia and an increased incidence of lymphoma.

Meningitis is a rare but potentially life threatening complication which has been reported in patients treated with anti-TNF therapy. Listeria monocytogenes infection is frequently seen, and cases of meningitis secondary to this agent have been reported in both infliximab and etanercept therapy [5,6]. Other reported cases of meningitis associated with etanercept have been secondary to tuberculosis, pneumococcus, varicella zoster and cryptococcus [4,7-10]. Although there have been case reports of aseptic [11], viral, bacterial and fungal meningitis associated with infliximab treatment, this case is the first in the literature to our knowledge reporting aseptic meningitis in a patient on etanercept.

## Case presentation

A 53 year-old Caucasian lady was admitted to the Medical Assessment Unit with a 4 day history of a spiking pyrexia, a dry macular erythematous rash and a new onset of confusion, generalised lethargy and difficulty sleeping.

Her past medical history included childhood rheumatic fever, epilepsy (completely controlled on cambamazepine), and rheumatoid arthritis, for which, 2 months previously, she had commenced weekly subcutaneous etanercept (50 mg), having failed to respond well to methotrexate and hydroxychloroqine alone. She had suffered a number of flare-ups in the proceeding 12 months, requiring 3 intramuscular steroid injections for symptomatic control. On admission, she was also taking tramadol, ibuprofen and 5 mg of folic acid. She is a full time carer for her disabled husband, and smokes 4 cigarettes daily.

Initial investigations revealed abnormal liver function (ALT 204 iu/L, ALP 393 iu/L), a urine dipstick positive for leucocytes and nitrites and a low white blood cell count of 3.9 × 10^9^/L with raised inflammatory markers. She was afebrile and haemodynamically stable with a normal chest x-ray, and so was treated for a presumed urinary tract infection with trimethoprim. An abdominal ultrasound showed slight fatty hepatic infiltration, and urine cultures gave mixed growth.

Over the next 3 days there was a general deterioration in her condition, becoming increasingly confused with a GCS of 12–13/15, and a pyrexia of up to 38.5°C. Despite conversion of her antibiotics to intravenous amoxicillin, her GCS fell to 10 and she became severely tachypnoeic and peripherally shut down. With an evolving sepsis apparent, she was fluid resuscitated and normal CT head permitted a lumbar puncture. She was transferred to the intensive care department for further management. The CSF analysis showed a raised white cell count of 130/μL (50% polymorphs), a glucose of 1.4 mmol/L and a protein of 1.02 g/L. This was highly suggestive of bacterial meningitis, despite no meningism clinically, so she was started on intravenous ceftriaxone and acyclovir (to cover HSV encephalitis). Subsequent CSF culture revealed no growth, and PCR was negative for cryptococcus, meningococcus, listeria and common viral causes (HSV, VZV and enterovirus). Her confusion and clinical parameters improved and 3 days later she was transferred from ITU. Although slight confusion persisted for some days, she completed her course of IV antibiotics and was discharged home after a further 10 days.

## Discussion

This case highlights a very rare but serious complication of a now commonly used medication, both within rheumatology and increasingly elsewhere. Having failed to achieve adequate disease control on non-biological therapy, a difficult future management dilemma exists – whether patients who have experienced serious infections should be re-trialled on anti-TNF therapy, or whether the risk of developing another life threatening complication outweighs the potential benefit. This case also highlights a previously unreported occurrence of aseptic meningitis in a patient taking etanercept, and although this patient had received antibiotics prior to the lumbar puncture sample, CSF PCR for all previously reported bacterial causes of meningitis in such patients was negative, suggesting either a novel microbial agent or a true aseptic meningitis.

## Consent

Written informed consent was obtained from the patient for the publication of this case report. A copy of the written consent is available for review by the Editor-in-Chief of this journal.

## Competing interests

The authors declare that they have no competing interests.

## Authors' contributions

JF and MB principally authored the manuscript. SS was a major contributor to data collection. All authors have read and approved the final manuscript.
